# Internal Fat and Cardiometabolic Risk Factors Following a Meal-Replacement Regimen *vs.* Comprehensive Lifestyle Changes in Obese Subjects

**DOI:** 10.3390/nu7125500

**Published:** 2015-12-01

**Authors:** Daniel König, Denise Zdzieblik, Peter Deibert, Aloys Berg, Albert Gollhofer, Martin Büchert

**Affiliations:** 1 Department for Nutrition, Institute for Sports and Sports Science, University of Freiburg, Schwarzwaldstr. 175, 79117 Freiburg, Germany; Daniel.koenig@uniklinik-freiburg.de (D.K.); Denise.Zdzieblik@sport.uni-freiburg.de (D.Z.); berg.aloys@web.de (A.B.); AG@sport.uni-freiburg.de (A.G.); 2 Department of Exercise and Sports Medicine, Centre for Internal Medicine, University Hospital Freiburg, Hugstetterstr. 175, 79106 Freiburg, Germany; Peter.Deibert@uniklinik-freiburg.de; 3 Department of Radiology—Medical Physics, University Medical Center Freiburg, Breisacher Straße 60a, 79106 Freiburg, Germany; Martin.Buechert@uniklinik-freiburg.de

**Keywords:** meal replacement, visceral fat, metabolic syndrome, lifestyle intervention

## Abstract

The aim of the present study was to investigate the effect of a meal-replacement regimen *vs.* comprehensive lifestyle changes in overweight or obese subjects on intra-abdominal fat stores (Magnetic Resonance Imaging (MRI) measurements) and cardiometabolic risk factors. Forty-two obese men (*n* = 18) and women (*n* = 24) (age 49 ± 8 years; weight 96.3 ± 12.1 kg; BMI 32.7 ± 2.3 kg/m^2^) were selected for this randomized parallel-group design investigation. Subjects in the lifestyle group (LS-G; *n* = 22) received dietary counselling sessions and instructions how to increase physical activity. In the meal replacement group (MR-G; *n* = 20) meals were replaced by a low-calorie drink high in soy protein. After six months, subjects in the LS-G lost 8.88 ± 6.24 kg and subjects in the MR-G lost 7.1 ± 2.33 kg; *p* < 0.01 for changes within groups; no significant differences were found between the groups. Lean body mass remained constant in both intervention groups. MRI analyses showed that internal fat was significantly reduced in both groups to a comparable amount; the higher fat loss in the LS-G in the abdominal area was due to a higher reduction in subcutaneous fat. Both interventions significantly reduced components of the cardiometabolic risk profile and leptin levels. The decrease in the adipokines fetuin A and resistin was more pronounced in the MR-G. In conclusion, both interventions significantly reduced body weight, total fat mass and internal abdominal fat while preserving lean body mass. The reduction in the adipokines fetuin A and resistin was more pronounced in the meal replacement group suggesting an additional effect of soy protein components.

## 1. Introduction

Hypercaloric diets and sedentary behaviour are cornerstones in the development of obesity, the metabolic syndrome and type 2 diabetes mellitus. Hence, comprehensive lifestyle changes improve both, weight loss and metabolic risk factors.

Several lines of evidence suggest that one of the most important goals for lifestyle interventions is the reduction in fat mass and in particular the reduction in intra-abdominal/internal fat. It has been shown that cardiometabolic risk factors increase as a function of visceral fat accumulation [1]. A higher amount of visceral fat—relative to subcutaneous fat—is related to extra-adipocyte fat storage, reduced insulin sensitivity and increased pro-inflammatory adipokine concentrations. Therefore, the appropriate measurement of the amounts of visceral fat and the ratio of visceral/subcutaneous fat is very important for the evaluation of the pathophysiological impact of total fat stores. Several measures of body composition routinely used in the clinic (e.g., waist circumference, waist/hip ratio or bioimpedance) have failed to predict visceral fat mass accurately [1].

Lifestyle interventions are effective in significantly reducing the amount of visceral fat. However, there is still an ongoing discussion which kind of lifestyle alteration is associated with the highest decrease in visceral fat and thus with the greatest benefit with respect to cardiometabolic risk factors [2,3,4].

The rational background for the present investigation was that although MR improve body weight and cardiometabolic risk factors [3,5,6], data on the respective effects on different fat stores is not available. MR regimens are criticized as associated with a loss in muscle mass favouring weight regain and that very low calorie diets predominantly reduce visceral fat in the early phase of weight loss interventions [4]. Moreover, little is known if the effects of a comprehensive lifestyle intervention (LSI) including aerobic exercise, psychological coaching and dietary intervention are superior to a MR regimen, particularly with respect to the influence on visceral fat [2].

## 2. Methods

In the present study, the effects of a six months meal replacement regimen (MR) on body composition and in particular on the amount of visceral and subcutaneous fat were investigated. In addition, body weight, metabolic risk factors and several adipokine concentrations were determined after six weeks and six months in order to document both short and longer term metabolic effects of the intervention. The results were compared to the respective effects of a comprehensive lifestyle intervention (LSI).

### 2.1. Subjects

Forty-two obese men (*n* = 18) and women (*n* = 24) (age 49 ± 8 years; weight 96.3 ± 12.1 kg; BMI 32.7 ± 2.3 kg/m^2^) were recruited from the outpatient database of the University Hospital for this randomized parallel-group design investigation. The participants should be between the age of 18 and 65 years (27 ≤ BMI ≤ 40) and capable to carry out a six month lifestyle intervention including physical exercise training.

Subjects with type 2 diabetes mellitus, clinically significant illnesses or patients who took anti-diabetic or lipid-lowering drugs were excluded. All subjects completed a comprehensive medical examination and routine blood tests. Written informed consent was provided by all subjects, and the study protocol was approved by the Ethical Committee of the University of Freiburg. Subjects were randomized into two equal groups as described previously using a random list [7]. The data presented here represent the pooled results of two studies that were performed identically in a comparable population group. The randomization process was done for each study separately and the pooling took place after completion of each study.

### 2.2. Intervention Program

The intervention in the lifestyle group (LS-G) consisted of 10 weekly teaching sessions related to nutrition, physical exercise and motivation. All sessions were held by certified experts in their respective fields. In addition, subjects received a hand-out with dietary advice and recommendations for lifestyle changes that were in accordance with the “German Society of Nutrition“ and the ”German Society of Sports Medicine and Prevention“ [8]. The prescribed diet was a moderate-fat, nutrient-balanced weight reduction diet consisting of 1200 to 1500 kcal per day for women and 1500 to 1800 kcal per day for men, with approximately 50–55 percent of the calories coming from carbohydrates, preferably with a low GI, 25–30 percent from fat, and 15–20 percent from protein. Dietary behaviour was checked using two 24-h dietary recalls that were used for dietary compliance and that were individually discussed at the nutritional teaching sessions.

The subjects in the LS-G were instructed to increase physical activity according to the guidelines of the “German Society of Sports Medicine”. They performed three physical activity sessions per week with an intensity ranging between 55% to 75% of VO_2_max. In the first six weeks, physical activity was performed as a group session supervised by a physical education teacher two times/week; thereafter, the group sessions took place once a week. Participants were instructed to perform the other physical activity sessions independently.

The subjects assigned to the meal replacement group (MR-G) were instructed to replace two daily meals with a commercially available soy-yoghurt-honey preparation (Almased^®^) for the first six weeks. During the following 20 weeks, only one daily meal was replaced by the preparation. The dietary intake of fat during this second phase was not to exceed 60 g per day. The first six-week diet contained about 1000 kcal per day for women and 1200 kcal for men, and then, in the following 20 weeks, aimed at a maximum of 1500 kcal for women and 1700 kcal for men.

The data collected at enrolment and after six, and 26 weeks were body weight, waist and abdominal circumference, self-reported medical history, blood pressure, glucose, serum lipids and plasma levels of several adipokines. Leptin, resistin and fetuin A were measured by commercially available ELISA kits (DSL Deutschland GmbH, Sinsheim, Germany). All other laboratory analyses were done in the central laboratory of the University hospital using clinical routine methods. Waist circumference was taken with a non-distensible tape measure according to published guidelines [9]. At baseline and after 26 weeks, body composition analyses using the technique of air displacement plethysmography were performed (Bod Pod^®^, [10]).

### 2.3. MRI Measurements

MRI (Magnetic Resonance Imaging) measurements of total abdominal fat and the amount of subcutaneous and internal fat were determined at baseline and after 26 weeks. MRI measurements were performed on a 1.5 T short bore, whole-body MRI system with an inner diameter of 70 cm (Magnetom Espree, Siemens Healthcare, Erlangen, Germany). Data were acquired using a gradient echo based Dixon sequence as described previously [11]. The region of interest was covered by a multi-array spine coil in combination with two multi-array body coils.

Fat/Water MRI data were analysed after a two-point-Dixon fat water image reconstruction using an active contour snake segmentation to separate subcutaneous and internal adipose tissue, including visceral adipose tissue, muscular fat and bone marrow [11]. The classification follows the one used by Shen [12] and was done for the abdominal region ranging from the highest cranial extension of the liver (exhaled position) to the first cranial slice displaying the femoral heads.

### 2.4. Statistics

Normality of all variables was tested before statistical analyses using the Kolmogorov-Smirnov test procedure. Testing for changes between the examinations within the intervention groups was performed by applying the paired two-sample *T*-test. Multiple testing was considered using the Holm-Bonferroni method. Testing for changes between groups following the intervention (LS-G *vs.* MR-G) was done by using two-way repeated-measures analysis of variance (ANOVA) for continuous variables. The factors were treatment group (LS-G *vs.* MR-G) and time (levels were pre and post intervention (6 and 26 weeks).

All P values were two-sided and a *P* value of 0.05 or less was considered to indicate statistical significance. Analysis was conducted with the use of SPSS software (version 20.0.1, IBM, Armonk, NY, USA).

## 3. Results

As stated before, the data represent the pooled results of two studies that were performed in an absolute identical manner. In total, from the outpatient database of the University Hospital, 117 potentially eligible subjects were contacted and asked concerning exclusion criteria. Seventy-five subjects were invited for screening and 50 subjects were eligible and randomized into the LS-G or MR-G. Forty-two patients completed the study and attended at least 75% of the meetings/training sessions. Eight participants dropped out: One subject changed the residence, five had claustrophobia, one subject was dissatisfied with the randomization outcome and one did not show-up again for follow-up for unknown reason.

For the final analysis, 22 subjects in the lifestyle group (LS-G) and 20 subjects in the meal replacement (MR-G) group were included.

### 3.1. Anthropometric Parameters

**Figure 1:**
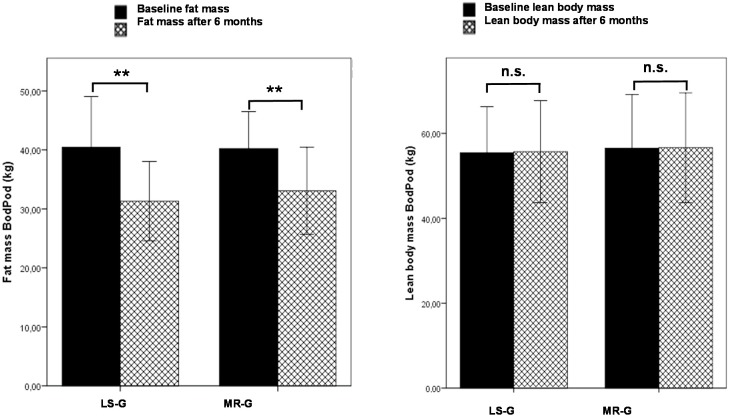
Changes in fat mass (PodBod) and lean body mass (PodBod) after six months by either lifestyle changes (LS-G) or a meal replacement regimen (MR-G); black bar, baseline values; grey hatched bars, post-interventional values after six months. (Error bars: ±1 standard deviation; *, *p* < 0.05; **, *p* < 0.01, n.s., not significant).

**Figure 2:**
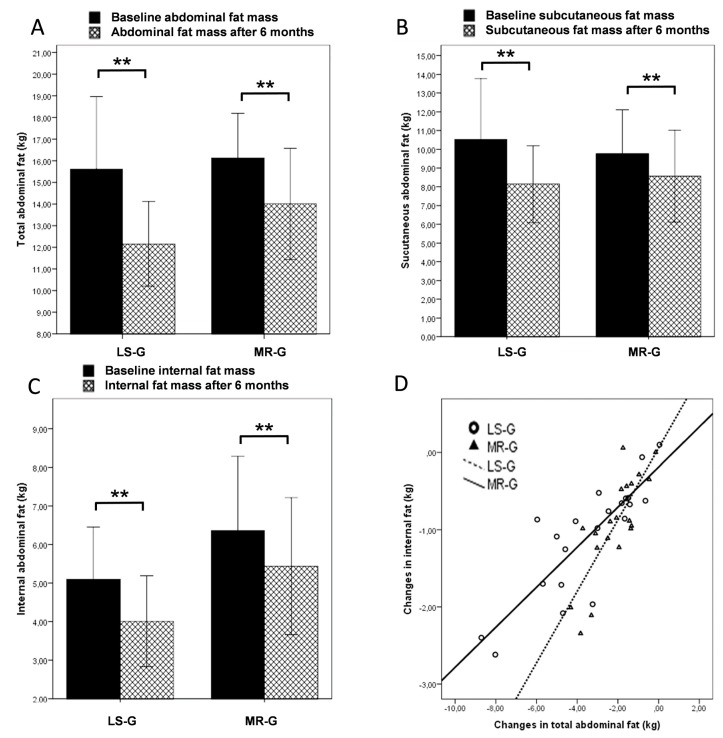
MRI findings: Changes in total abdominal fat mass (**A**); subcutaneous abdominal fat (**B**); internal abdominal fat (**C**) and the correlation between changes in total abdominal fat and internal abdominal fat in dependence of the intervention group (lifestyle changes LS-G or meal replacement regimen MR-G); black bars, baseline values; grey hatched bars, post-interventional values after six months. (Error bars: ±1 standard deviation; *, *p* < 0.05; **, *p* < 0.01).

The changes in weight and BMI are shown in Table 1. After six weeks, the decrease in weight and BMI was more distinct in the MR group whereas after six months, weight loss was more pronounced in the LS group. At the end of the study, subjects in the LS-G had lost 8.88 ± 6.24 kg and subjects in the MR-G had lost 7.1 ± 2.33 kg. The changes within each group were highly significant (*p* < 0.01) whereas no significant differences could be observed between the groups. Figure 1 shows the results from the Bod Pod analysis indicating that the higher weight loss in the LS-G was due to a higher decrease in fat mass. Lean body mass did not change in either of the intervention groups. Figure 2 shows the results of the MRI scans. In the abdominal region, subjects in the LS group lost more fat than the MR group (A); however, the differences between the intervention groups were not significant for all MRI measures. Subjects in the MR-G exhibited lower subcutaneous and higher internal abdominal fat in the abdominal region at baseline. The latter findings could be explained by a higher proportion of females in the LS-G compared to the MR-G. However, although the female subjects in this investigation exhibited a higher mean subcutaneous fat mass (11.4 kg) than the male participants (8.6 kg), there were no distinct gender-related changes in the course of the intervention that could explain the different outcome in the LS-G *vs.* MR-G. Both, female and male subjects lost 2.9 kg of total abdominal fat and the surplus amount of subcutaneous fat loss was only 300 g in the female participants. In the LS-G, the surplus in subcutaneous fat loss was 1.4 kg (B) while internal fat loss was almost identical (C).

**Table 1:** Weight and BMI during the course of the study. Mean ± standard deviation (SD). LS-G, lifestyle intervention group; MR-G, meal replacement group. §, *p* < 0.05 compared to baseline; ◊, *p* < 0.01 compared to baseline (paired *T*-Test). Differences between the intervention groups (ANOVA) were not significant at any point of the investigation.

		Baseline	6 Weeks	6 Months	Changes after 6 Weeks	Changes after 6 Months
		Mean (SD)	Mean (SD)	Mean (SD)	Mean (SD)	Mean (SD)
Weight (kg)	**LS-G**	95.9 (12.01)	92.1 (11.9) ^§^	87.0 (12.2) **^◊^**	−3.77 (2.85)	−8.88 (6.24)
	**MR-G**	96.7 (12.6)	91.3 (12.8) **^◊^**	89.7 (13.1) **^◊^**	−5.42 (1.86)	−7.06 (2.33)
BMI (kg/m^2^)	**LS-G**	32.5 (2.65)	31.2 (2.62) ^§^	29.4 (2.46) **^◊^**	−1.28 (0.94)	−3.06 (2.13)
	**MR-G**	32.9 (1.88)	31.0 (2.08) **^◊^**	30.5 (2.43) **^◊^**	−1.85 (0.59)	−2.41 (0.77)

Per kg fat mass lost during the intervention, participants in the MR-G lost more abdominal fat which is further illustrated by the scatterplot and the linear equation (D).

### 3.2. Cardiometabolic Risk Factors

Although there were no significant differences between the intervention groups at any time of the study, there were discrepant courses of parameters and differences in the significance levels within the two groups (Table 2). Total cholesterol decreased significantly after six weeks in both groups and rose again to near baseline levels after six months. Triglycerides levels were initially higher in the MR-G and decreased in both groups after six weeks; after six months triglycerides further decreased in the MR-G but rose again in the LS-G. After six month, triglycerides dropped by 51.4 ± 93.1 mg/dL in the MR-G and 19.4 ± 49.2 mg/dL in the LS-G. LDL-Cholesterol dropped significantly in both groups after six weeks with a more pronounced decline in the MR-G. After six months LDL-levels largely returned to baseline in both groups. Initial levels of glucose and HbA1c were higher in subjects in the LS-G. Glucose levels dropped in both groups but the decrease was only significant in the lifestyle group. HbA1c also dropped in both groups and after six months, the changes were significant in both groups. The changes were more distinct in the LS-G, however, comparable to glucose levels, initial HbA1c was also higher in subjects in the lifestyle group.

**Table 2:** Total cholesterol, triglycerides, LDL- and HDL-cholesterol, glucose and HbA1c levels during the course of the study. Mean ± standard deviation (SD). LS-G, lifestyle intervention group; MR-G, meal replacement group. §, *p* < 0.05 compared to baseline; ◊, *p* < 0.01 compared to baseline (paired *T*-Test). Differences between the intervention groups (ANOVA) were not significant at any point of the investigation.

		Baseline	6 Weeks	6 Months	Changes after 6 Weeks	Changes after 6 Months
		Mean (SD)	Mean (SD)	Mean (SD)	Mean (SD)	Mean (SD)
Cholesterol (mg/dL)	**LS-G**	225 (37.4)	202 (39.9) **^◊^**	215 (33.4)	−23.7 (31.3)	−11.3 (30.1)
	**MR-G**	228 (38.5)	195 (31.4) **^◊^**	223 (41.4)	−32.3 (24.3)	−4.71 (31.5)
Triglycerides (mg/dL)	**LS-G**	138 (61.4)	103 (37.4) **^◊^**	118 (60.5)	−34.8 (51.1)	−19.4 (49.2)
	**MR-G**	172 (115)	125 (45.4) **^◊^**	120 (41.9) **^◊^**	−46.9 (88.2)	−51.4 (93.1)
LDL-Cholesterol (mg/dL)	**LS-G**	140.2 (40.4)	131 (34.6) ^§^	139 (31.6)	−8.9 (35.3)	−0.51 (31.3)
	**MR-G**	147.2 (31.7)	127 (27.5) **^◊^**	150 (36.1)	−20.3 (17.9)	3.27 (24.7)
HDL-Cholesterol (mg/dL)	**LS-G**	53.4 (14.7)	48.8 (8.99) **^◊^**	54.6 (13.2)	−4.53 (10.9)	1.19 (9.17)
	**MR-G**	50.1 (9.42)	47.4 (7.87) ^§^	52.3 (9.52) **^◊^**	−2.65 (6.76)	2.25 (7.29)
Glucose (mg/dL)	**LS-G**	100.7 (25.1)	91.9 (14) ^§^	92.2 (15.9) **^◊^**	−8.70 (17.3)	−8.48 (15.1)
	**MR-G**	95.2 (19.9)	93.4 (11.9)	90.2 (10.6) ^#^	−1.35 (10.7)	−4.55 (13.8)
HbA1c (%)	**LS-G**	5.64 (0.52)	5.51 (0.44) **^◊^**	5.51 (0.38) **^◊^**	−0.13 (0.23)	−0.13 (0.26)
	**MR-G**	5.58 (0.55)	5.52 (0.57)	5.50 (0.42) ^§^	−0.06 (0.18)	−0.07 (0.34)

### 3.3. Adipokine Levels

None of the adipokines showed significant differences between the groups (Table 3). Plasma leptin levels decreased significantly and to a comparable degree in both groups. The reduction in resistin and fetuin A levels was more pronounced and only significant in the MR-G.

**Table 3:** Plasma levels of the adipokines Leptin, Resistin and Fetuin A during the course of the study. Mean ± standard deviation (SD). LS-G, lifestyle intervention group; MR-G, meal replacement group. §, *p* < 0.05 compared to baseline; ◊, *p* < 0.01 compared to baseline (paired *T*-Test).Differences between the intervention groups (ANOVA) were not significant at any point of the investigation.

		Baseline	6 Weeks	6 Months	Changes after 6 Weeks	Changes after 6 Months
		Mean (SD)	Mean (SD)	Mean (SD)	Mean (SD)	Mean (SD)
Leptin (µg(L)	**LS-G**	19.1 (11.9)	12.7 (8.39) **^◊^**	9.85 (8.02) **^◊^**	−7.91 (10.0)	−10.3 (11.3)
	**MR-G**	18.7 (19.3)	11.6 (10.9) **^◊^**	9.36 (6.97) **^◊^**	−5.70 (10.7)	−8.47 (13.2)
Resistin (µg/L)	**LS-G**	4.76 (1.68)	5.03 (1.58)	4.85 (1.88)	0.06 (0.78)	−0.13 (1.12)
	**MR-G**	5.53 (2.44)	4.67 (1.38) ^§^	4.51 (1.35) **^◊^**	−0.88 (1.64)	−0.88 (1.43)
Fetuin A (µg/mL)	**LS-G**	0.41 (0.1)	0.41 (0.1)	0.39 (0.09)	0.01 (0.08)	−0.01 (0.08)
	**MR-G**	0.38 (0.07)	0.36 (0.08) **^◊^**	0.35 (0.08) ^§^	−0.03 (0.06)	−0.03 (0.04)

## 4. Discussion

The main finding of the present study was that the meal replacement regimen decreased internal abdominal fat stores to the same degree as a comprehensive lifestyle intervention. In addition, total fat loss was not significantly different between the intervention groups, and both groups showed no decrease in fat free mass. The results regarding reductions in body weight and fat mass are in keeping with previous studies investigating the effect of meal replacements or comprehensive lifestyle interventions in overweight or obese subjects [13,14]. This investigation did not confirm the findings of a review by Chaston *et al.* that the preferential loss of internal fat by very low calorie diets disappears when the intervention period is longer than four weeks [4].

Nevertheless, albeit not significant, the lifestyle intervention further reduced total and abdominal fat stores. The results from the MRI-scans suggest that the additional fat loss in the lifestyle group could mainly be attributed to an increased fat loss from subcutaneous fat stores. This could not be explained by the higher proportion of women in the LS-G, since the gender-related differences following the intervention were small compared to the differences between the meal replacement and the lifestyle intervention. Although the evidence cannot be directly deducted from the design of the present investigation, it could be speculated that the greater weight loss is related to the additional training program in the lifestyle group. Previous studies have also shown that additional exercise did not further increase intra-abdominal fat loss when added to a hypocaloric diet [15,16].

However, although the individual amount and the intensity of physical exercise was supervised by the sports instructors, the exact duration and intensity of individual sports activities was not monitored.

Both interventions significantly reduced the metabolic risk profile. From a physician’s perspective, the findings after six months had more relevance than the results from six weeks. Regarding changes with clinical significance, the reduction in triglycerides and increase in HDL-cholesterol was more pronounced in the meal replacement group whereas glucose and HbA1c-levels were lower in the lifestyle group. The latter finding could be due to higher baseline levels in the LS-G. Previous investigations have shown that meal replacement regimens induce rapid improvements in metabolic risk factors in subjects with the metabolic syndrome [17,18]. In the present investigation, the metabolic risk profile was relatively low, therefore, the improvements were also relatively small. However, the changes in both groups demonstrated that improvements could be accomplished and that the magnitude of beneficial effects increased as a measure of baseline levels.

Adipokine levels showed no significant differences between the groups during the course of the intervention. These adipokines were selected because they reflect the body’s fat stores (leptin) and it has been speculated that they play an important role in the initiation and propagation of the pro-inflammatory state associated with extra-adipocyte fat storage, insulin resistance, internal fat accumulation and atherosclerosis.

Leptin levels were comparably reduced in both groups whereas the reduction in resistin and fetuin A were more pronounced in the subjects in the MR-G. Although the levels of these adipokines were positively correlated with total fat mass and cardiometabolic risk factors (data not shown), the decrease was not directly correlated with a reduction in internal abdominal fat mass. It could be speculated that the higher reduction in fetuin A and resistin could be explained by the specific effects of soy protein. Some, albeit not all investigations have suggested a positive influence of soy protein, and in particular soy isoflavones, on proinflammatory adipokines, insulin resistance and body weight [19,20,21]. However, it has to be acknowledged that the design of the present investigation was not appropriate to establish a cause-effect relationship.

## 5. Conclusions

In conclusion, results from the present study have shown that both, lifestyle intervention by increased physical activity/hypocaloric low fat diet and meal replacement using a soy protein formula significantly decreased body weight, total fat mass and internal abdominal fat to a comparable amount while completely preserving lean body mass. Both interventions were associated with an improvement in metabolic risk factors although the effect of the intervention seemed to be dependent on pre-interventional levels. Although the effects in both groups were comparable with respect to internal abdominal fat mass, the reduction in adipokine levels was more pronounced in the meal replacement group suggesting an additional effect of soy protein related compounds e.g., genistein or isoflavones. This, however, needs to be further examined in forthcoming studies. It is important to emphasize that the long-term process of atherosclerosis cannot be influenced by short-term dietary modification. All interventions in obese subjects, particularly in patients with metabolic risk factors and increased atherosclerotic burden, should aim at inducing long-term modifications and not transient alterations in both, weight management and atherosclerotic risk factors.
